# Postoperative controlling nutritional status score is an independent risk factor of survival for patients with small hepatocellular carcinoma: a retrospective study

**DOI:** 10.1186/s12893-021-01334-9

**Published:** 2021-09-07

**Authors:** Wei Peng, Minghong Yao, Kang Zou, Chuan Li, Tianfu Wen, Xin Sun

**Affiliations:** 1 Department of Liver Surgery, West China Hospital, Sichuan University, Chengdu, 610041 China; 2 Chinese Evidence-based Medicine Center, West China Hospital, Sichuan University, Chengdu, 610041 China; 3 Laboratory of Liver Transplantation, West China Hospital, Sichuan University, Chengdu, 610041 China

**Keywords:** Controlling nutritional status, Hepatocellular carcinoma, Milan criteria, Liver resection

## Abstract

**Background:**

The controlling nutritional status (CONUT) score has been widely used to evaluate the nutritional and immunological status. Clinical value of postoperative CONUT (PoCONUT) score in hepatocellular carcinoma (HCC) remains unknown. This study assessed whether PoCONUT score could serve as a useful predictor of survival for patients with small HCC.

**Methods:**

547 consecutive patients with small HCC who underwent liver resection between February 2007 and December 2015 were included in this retrospective case-control study. Patients were categorized into two groups: low PoCONUT group (PoCONUT score ≤ 2, n = 382) and high PoCONUT group (PoCONUT score ≥ 3, n = 165). Propensity score matching (PSM) analysis was applied to balance the bias in baseline characteristics. A cumulative survival curve was established by the Kaplan–Meier method, and differences in OS and RFS among CONUT score groups were determined by the log rank test. Cox proportional hazard regression analysis was used to evaluate the association of PoCONUT score and overall survival (OS) and recurrence-free survival (RFS), with calculation of hazard ratios (HRs) and 95 % confidence intervals (95 % CIs).

**Results:**

Cox proportional hazard regression analysis suggested that the PoCONUT score was an independent risk factor for both OS and RFS in patients with small HCC before and after PSM.

**Conclusions:**

High PoCONUT score helps to predict worse OS and RFS in patients with small HCC who underwent liver resection.

## Background

HCC is one of the most common malignancies and remains the third leading cause of cancer-related deaths worldwide [1]. Owing to the high prevalence of hepatitis B virus (HBV), nearly half of the new cases and HCC related deaths occurred in China [2]. Liver resection is widely accepted as the standard treatment for patients with small HCC meeting Milan Criteria [3–5]. Despite the advances in perioperative management and postoperative prophylactic treatment, the overall prognosis of patients who underwent liver resection remains unsatisfactory [6]. Biological properties of tumor and remnant liver function were the main prognostic factors affecting overall survival and recurrence after liver resection [7, 8].

Recently, nutritional and immunological status was found to be related to the surgical prognosis of many kinds of malignancies [9, 10]. One of the widely used scoring systems for nutritional and immunological status is the CONUT score, which has the advantage of being readily available from three simple parameters: serum albumin concentration, total lymphocyte count and total cholesterol concentration in peripheral blood [11]. Furthermore, a high preoperative CONUT (PreCONUT) score was reported to be associated not only with postoperative complications but also with the long-term prognosis of patients with HCC who underwent liver resection [12, 13]. However, these studies only focus on the PreCONUT score, the clinical value of PoCONUT score, which may reflect the immunological and nutritional status after surgical removal of the tumor, is largely undefined.

This study was designed to evaluate whether PoCONUT score could serve as a useful prognostic predictor for OS and RFS of patients with small HCC who underwent liver resection.

## Method

### Patients

Clinical data were retrospectively retrieved for 547 consecutive patients with small HCC meeting Milan criteria who underwent liver resection as an initial treatment between February 2007 and December 2015 in the Department of Liver Surgery & Liver Transplantation Center of West China Hospital, Sichuan University. Diagnosis of HCC, microvascular invasion (MVI), tumor differentiation was assessed by a postoperative pathological examination. Liver cirrhosis was also determined in postoperative histopathology examination in accordance with the Ishak scoring system [14]. This study was conducted with the approval of the Ethics Committee of West China Hospital, Sichuan University and all methods were performed in accordance with the Declaration of Helsinki.

### Definition and cut-off value for CONUT score

Preoperative blood samples were taken within 1 week before surgery. CONUT score was calculated based on serum albumin concentration, total lymphocyte count and total cholesterol concentration in each patient (Table 1) [11]. In the present study, we defined CONUT score ≤ 2 as a “low CONUT” group, and CONUT score ≥ 3 as a “high CONUT” group as previously described [15, 16]. PoCONUT score was calculated based on results of postoperative blood samples at 1st follow-up visit, one month after the surgery.

**Table 1:** Assessment of undernutrition degree by CONUT score

Parameters	Undernutrition degree			
	Normal	Light	Moderate	Severe
Serum albumin (g/dl)	≥ 3.5	3.0–3.49	2.5–2.99	< 2.5
Score	0	2	4	6
Total lymphocytes/ml	> 1600	1200–1599	800–1199	< 800
Score	0	1	2	3
Cholesterol (mg/dl)	> 180	140–180	100–139	< 100
Score	0	1	2	3
Total score	0–1	2–4	5–8	9–12

*CONUT *controlling nutritional status

### Follow-up visit

Each patient was regularly followed up at the 1st and every 3 months within 3 years after surgery, and every 6 months thereafter. Follow-up examinations included blood cell and differential counts, alpha-fetoprotein (AFP) level, biochemical test combo or liver function test, HBV-DNA level (if the patient was diagnosed with HBV infection), image and physical examinations. When recurrence was suspected, additional examinations such as contrast-enhanced computed tomography (CT) and/or hepatic angiography were performed. Recurrent HCC were treated with salvage liver transplantation, repeat liver resection, radiofrequency ablation, transcatheter arterial chemoembolization or systemic therapy according to a strategy previous described [17]. RFS time was defined as the time interval between surgery and recurrence, while OS time was defined as the time interval between the surgery and death or last follow-up visit. The last follow-up date was the end of June 2016.

### Statistical analysis

Continuous variables were present as mean ± standard deviation or median (range) and were compared using independent sample t-test. Categorical data were present as a number (%) and analyzed using the chi-square test or Fisher’s exact test. A cumulative survival curve was established by the Kaplan–Meier method, and differences in OS and RFS among CONUT score groups were determined by the log rank test. Cox proportional hazard regression analysis was used to evaluate the association of CONUT score and OS and RFS, with calculation of HRs and 95 % CIs.

In this retrospective study, a PSM analysis was performed to minimize the selection bias between two groups. The possible clinicopathological variables (age, gender, total bilirubin, prothrombin time, hemoglobin, platelet, white blood cell, total cholesterol, PreCONUT score, tumor size, liver cirrhosis, MVI and tumor differentiation) were included in the PSM. Propensity scores were matched using a caliper of 0.1 and a neighbor matching algorithm was applied. A difference < 20 % of absolute value was acceptable [18, 19].

All statistical analyses were performed by SPSS software version 26.0 (SPSS company, Chicago, IL, USA). Calculated P values were two-sided, and a P value less than 0.05 was considered statistically significant.

## Results

### Baseline characters

The clinical and pathologic characters of the patients are summarized in Table 2. Of the 547 patients, 382 (69.8 %) had a PoCONUT score ≤ 2 (low PoCONUT group) and 165 (30.2 %) had a PoCONUT score ≥ 3 (high PoCONUT group). Patients in high PoCONUT group had higher total bilirubin and PreCONUT score, longer prothrombin time, lower hemoglobin, platelet, and white blood cell counts, they also tended to have HBV infection and liver cirrhosis (all P < 0.05). To minimize the difference between the two groups, a PSM analysis was applied. After this matching, 133 matched pairs were created. The baseline features were comparable between the two groups in the matched cohort (Table 3).

**Table 2:** Comparison of clinical and pathologic characters between two groups according to postoperative CONUT score in the whole study population

Variables	PoCONUT ≤ 2 N = 382	PoCONUT ≥ 3 N = 165	P
Age (years)	50.41 ± 12.62	49.16 ± 12.74	0.293
Male/female	330/52	134/31	0.153
HBsAg (+/**−**)	338/44	156/9	0.028
AFP > 400ng/ml	115 (30.1 %)	48 (29.1 %)	0.839
TBIL (µmol/L)	14.88 ± 6.13	16.53 ± 7.20	0.007
ALT (IU/L)	46.56 ± 37.08	44.79 ± 37.95	0.610
AST (IU/L)	39.26 ± 29.78	41.01 ± 29.70	0.530
PT (s)	12.09 ± 1.61	12.45 ± 1.20	0.011
CREA (µmol/L)	75.98 ± 14.94	76.61 ± 28.35	0.788
HGB (g/L)	145.81 ± 16.18	140.48 ± 18.59	0.001
PLT (*10^9/L)	126.43 ± 54.57	97.22 ± 38.91	< 0.001
WBC (*10^9/L)	5.57 ± 1.85	4.60 ± 1.64	< 0.001
Albumin (g/dL)	42.30 ± 4.68	41.92 ± 4.76	0.395
Total cholesterol (mg/dl)	163.59 ± 31.29	144.76 ± 27.34	< 0.001
PreCONUT ≤ 2	290 (75.9 %)	52(31.5 %)	< 0.001
Tumor size < 3 cm	207 (54.2 %)	74 (44.8 %)	0.050
Solitary tumor	341 (89.3 %)	145 (87.9 %)	0.658
Blood loss (mL)	337.70 ± 355.41	365.03 ± 270.57	0.377
Transfusion (+/**−**)	18/364	13/152	0.159
Operation duration (min)	237.16 ± 57.80	220.10 ± 62.72	0.162
Operation type major/minor	65/317	29/136	0.902
Cirrhosis (+/**−**)	307/75	151/14	0.001
MVI (+/**−**)	61/321	32/133	0.324
Poor differentiation	140 (36.7 %)	64 (38.8 %)	0.632
Hospital stay (d)	10.93 ± 5.12	11.71 ± 4.46	0.673

Data are displayed as mean ± standard deviation, or number of patients (percentage)

*CONUT* controlling nutritional status, *PreCONUT* preoperative CONUT, *PoCONUT* postoperative CONUT, *HBsAg* hepatitis B viral surface antigen, *AFP* alpha-fetoprotein, *TBIL* total bilirubin, *ALT* alanine aminotransferase, *AST* aspartate aminotransferase, *PT* prothrombin time, *CREA* creatinine, *HGB* hemoglobin, *PLT* platelet count, *WBC* white blood cell count, *MVI* microvascular invasion

**Table 3:** Comparison of clinical and pathologic characters between two groups according to postoperative CONUT score in propensity score matched pairs

Variables	PoCONUT ≤ 2 N = 133	PoCONUT ≥ 3 N = 133	P
Age (years)	51.71 ± 13.71	48.73 ± 12.33	0.058
Male/female	112/22	112/22	1.000
HBsAg (+/**−**)	123/10	124/9	1.000
AFP > 400ng/ml	39 (29.3 %)	42 (31.6 %)	0.790
TBIL (µmol/L)	15.90 ± 6.87	15.32 ± 6.17	0.473
ALT (IU/L)	47.20 ± 36.02	45.03 ± 37.64	0.631
AST (IU/L)	40.86 ± 23.18	39.77 ± 29.05	0.735
PT (s)	12.43 ± 2.33	12.38 ± 1.22	0.823
CREA (µmol/L)	76.03 ± 14.85	75.07 ± 16.50	0.616
HGB (g/L)	142.37 ± 17.45	142.61 ± 17.35	0.910
PLT (*10^9/L)	101.99 ± 41.30	103.24 ± 38.99	0.800
WBC (*10^9/L)	4.86 ± 1.74	4.80 ± 1.61	0.773
Albumin (g/dL)	41.55 ± 6.10	41.98 ± 4.39	0.461
Total cholesterol (mg/dl)	147.90 ± 28.77	145.90 ± 27.63	0.745
PreCONUT ≤ 2	51 (38.3 %)	50 (37.6 %)	1.000
Tumor size < 3 cm	72 (54.1 %)	63(47.4 %)	0.327
Solitary tumor	113 (85.0 %)	117 (88.0 %)	0.591
Blood loss (mL)	355.64 ± 317.88	358.12 ± 273.78	0.946
Transfusion (+/**−**)	9/124	9/124	0.159
Operation type major/minor	21/122	22/111	0.986
Operation duration (min)	226.46 ± 60.89	219.22 ± 65.56	0.389
Cirrhosis (+/**−**)	119/14	119/14	1.000
MVI (+/**−**)	27/106	27/106	1.000
Poor differentiation	58 (43.6 %)	48 (36.2 %)	0.260
Hospital stay (d)	11.05 ± 5.07	11.67 ± 4.78	0.875

Data are displayed as mean ± standard deviation, or number of patients (percentage)

*CONUT* controlling nutritional status, *PreCONUT* preoperative CONUT, *PoCONUT* postoperative CONUT, *HBsAg* hepatitis B viral surface antigen, *AFP* alpha-fetoprotein, *TBIL* total bilirubin, *ALT* alanine aminotransferase, *AST* aspartate aminotransferase, *PT* prothrombin time, *CREA* creatinine, *HGB* hemoglobin, *PLT* platelet count, *WBC* white blood cell count, *MVI *microvascular invasion

### Impact of PreCONUT score on OS and RFS

**Fig. 1:**
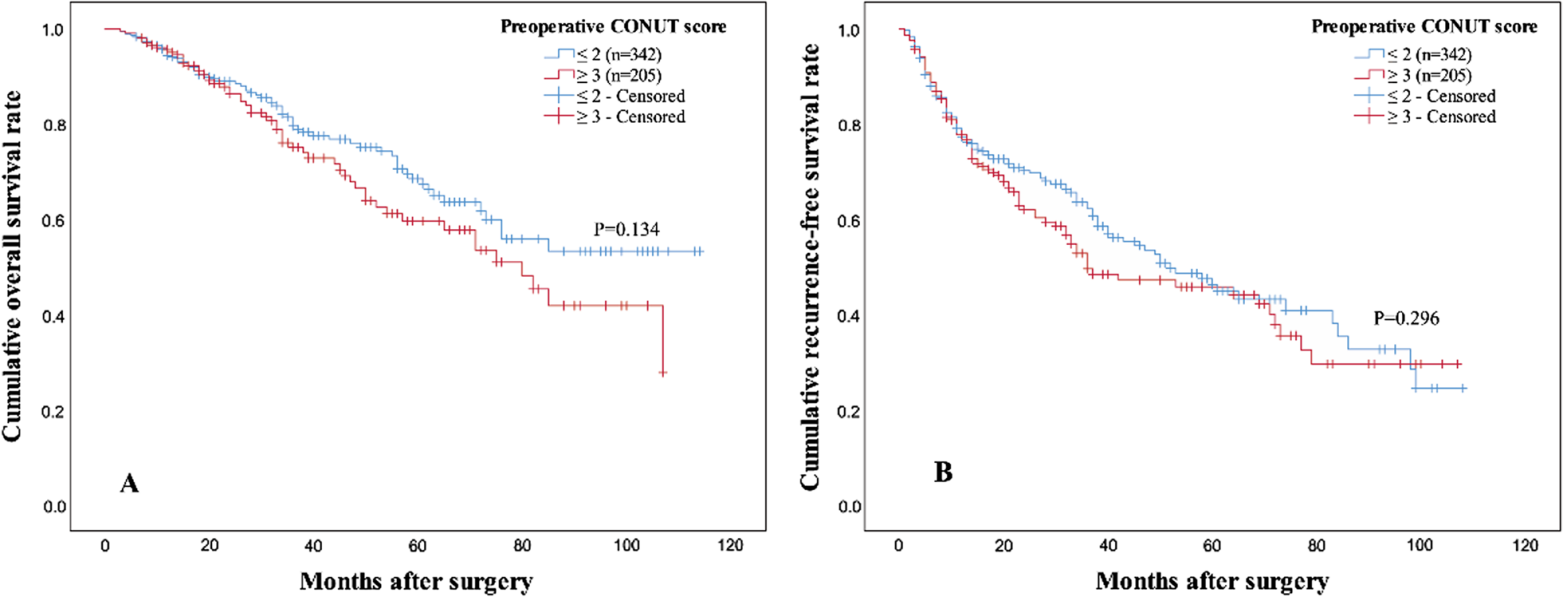
Kaplan–Meier curves of OS (**A**) and RFS (**B**) for patients in high and low preoperative CONUT score groups. There was no difference in cumulative OS or RFS rates of patients with  a high and low preoperative CONUT score. *OS* overall survival, *RFS* recurrence-free survival, *CONUT* controlling nutritional status

After a median follow-up time of 34 months, 223 (40.8 %) patients were found recurrent and 128 (23.4 %) patients died. 1-, 3-, 5-year estimated OS rates of patients in low PreCONUT group were 94.8 %, 80.2 and 68.8 % respectively, and 95.4 %, 74.9 and 59.2 % respectively for patients in high PreCONUT group (log-rank test, P = 0.134, Fig. 1A). 1-, 3-, 5-year estimated RFS rates of patients in low PreCONUT group were 78.8 %, 62.5 and 46.5 % respectively, and 77.4 %, 50.3 and 46.2 % respectively for patients in high PreCONUT group (log-rank test, P = 0.296, Fig. 1B).

### Impact of PoCONUT score on OS and RFS

**Fig. 2:**
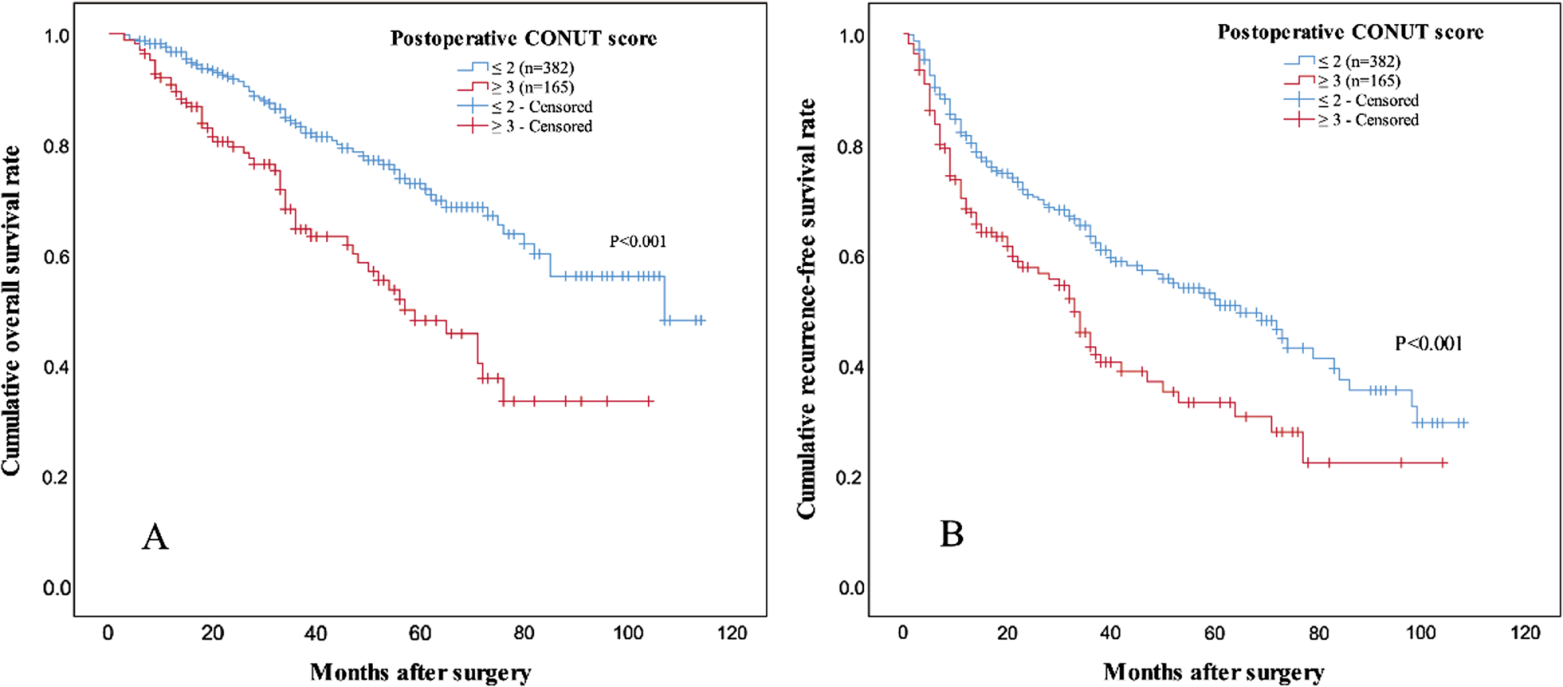
Kaplan–Meier curves of OS (**A**) and RFS (**B**) for patients with a high or low postoperative CONUT score before propensity score matching. Patients with a high postoperative CONUT score had a decreased OS and RFS rate. *OS* overall survival, *RFS* recurrence-free survival, *CONUT* controlling nutritional status

Before PSM, the 1-, 3-, 5-year estimated OS rates of patients in low PoCONUT group were 96.7 %, 83.6 and 72.8 % respectively, and 91.3 %, 64.7 and 48.2 % respectively for patients in high PoCONUT group (log-rank test, P < 0.001, Fig. 2 A). 1-, 3-, 5-year estimated RFS rates of patients in low PoCONUT group were 81.2 %, 63.3 and 51.4 % respectively, and 68.3 %, 43.4 and 33.4 % respectively for patients in high PoCONUT group (log-rank test, P < 0.001, Fig. 2B).

**Fig. 3:**
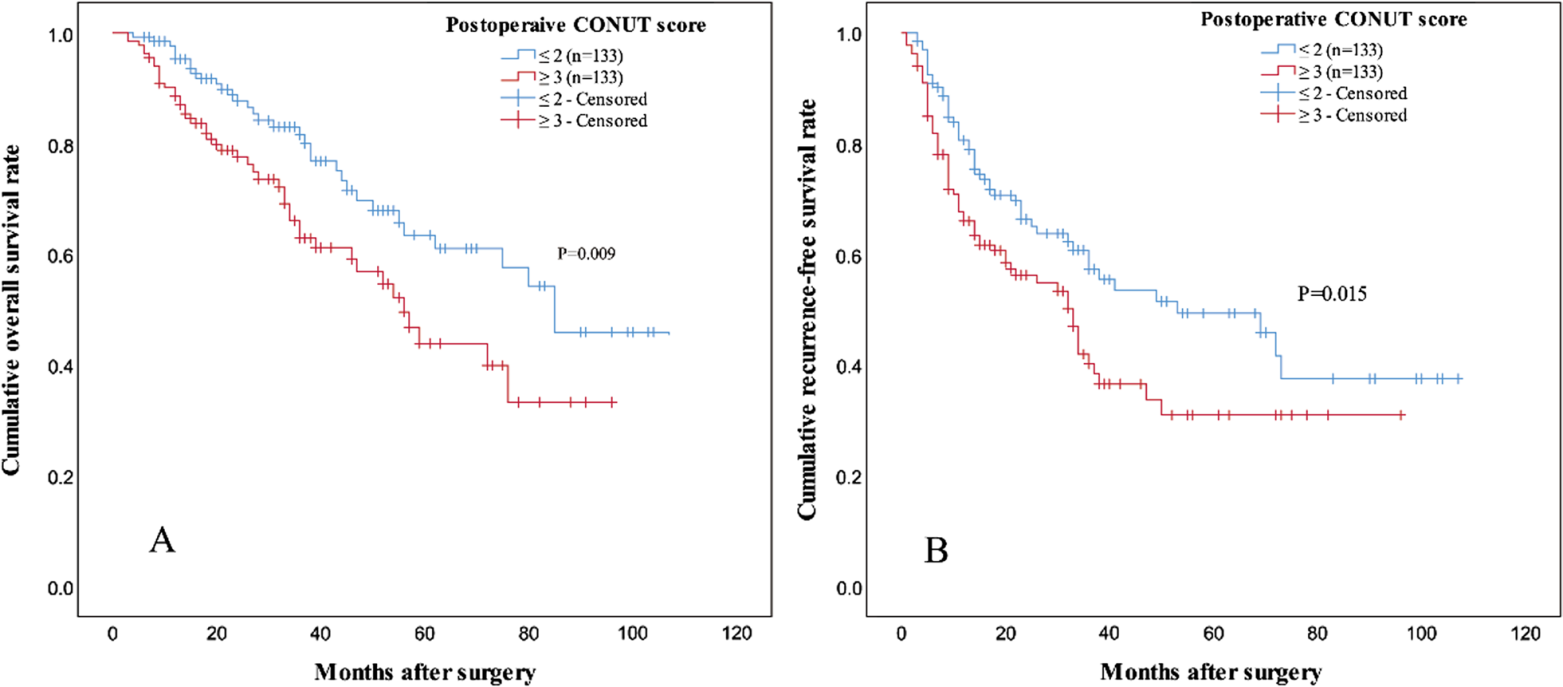
Kaplan–Meier curves of OS (**A**) and RFS (**B**) for patients with a high or low postoperative CONUT score after propensity score matching. Patients with a high postoperative CONUT score had a decreased OS and RFS rate. *OS* overall survival, *RFS* recurrence-free survival, *CONUT* controlling nutritional status

After PSM, 1-, 3-, 5-year estimated OS rates of patients in low PoCONUT group were 95.4 %, 81.2 and 63.3 % respectively, and 88.7 %, 63.0 and 44.2 % respectively for patients in high PoCONUT group (log-rank test, P = 0.009, Fig. 3A). 1-, 3-, 5-year estimated RFS rates of patients in low PoCONUT group were 80.4 %, 57.5 and 49.5 % respectively, and 66.1 %, 40.3 and 31.0 % respectively for patients in high PoCONUT group (log-rank test, P = 0.015, Fig. 3B).

### Risk factors of prognosis

Univariate and multivariate analyses were carried out to identify the risk factors of prognosis. As for OS, thirteen potential covariates with p values less than 0.10 in univariate analysis were further included in Cox regression analysis. And Cox regression analysis suggested that elevated AFP (P = 0.038, hazard ratio (HR) = 1.622, 95 % confidence interval (CI) 1.028–2.560), MVI (P = 0.007, HR = 1.929, 95 %CI 1.202–3.098) and PoCONUT score (P = 0.019, HR = 1.708, 95 %CI 1.091–2.676) were independent risk factors for reduced OS (Table 4).

**Table 4:** Univariate and multivariate analyses of prognostic factors for overall survival in a propensity score matched cohort

Factors	Univariate analysis		Multivariate analysis	
	HR (95 %CI)	P	HR (95 %CI)	P
Age	0.989 (0.969–1.009)	0.269		
Gender (male)	1.313 (0.695–2.481)	0.402		
HBsAg (+)	1.627 (0.512–5.168)	0.409		
AFP (> 400ng/mL)	1.694 (1.073–2.675)	0.024	1.622 (1.028–2.560)	0.038
TBIL	0.999 (0.960–1.040)	0.959		
ALT	1.010 (1.003–1.018)	0.007		
AST	1.010 (1.000–1.020)	0.052		
PT	1.069 (0.930–1.229)	0.346		
CREA	1.017 (0.999–1.035)	0.061		
HGB	1.003 (0.988–1.019)	0.663		
PLT	0.997 (0.991–1.004)	0.425		
WBC	0.942 (0.799–1.111)	0.476		
PreCONUT (≥ 3)	0.775 (0.499–1.204)	0.257		
Tumor size (3–5 cm)	0.970 (0.626–1.501)	0.890		
Tumor number (2–3)	1.589 (0.919–2.748)	0.097		
Blood loss	1.001 (1.000–1.002)	0.033		
Transfusion (+)	1.854 (0.921–3.735)	0.084		
Cirrhosis (+)	1.502 (0.607–3.720)	0.379		
Operation duration	1.078 (0.916–1.268)	0.367		
Operation type (major)	1.139 (0.797–1.626)	0.473		
MVI (+)	2.210 (1.390–3.514)	0.001	1.929 (1.202–3.098)	0.007
Poor differentiation (+)	1.287 (0.810–2.044)	0.285		
Hospital stay	1.060 (0.771–1.456)	0.720		
PoCONUT (≥ 3)	1.805 (1.155–2.820)	0.009	1.708 (1.091–2.676)	0.019

*CONUT* controlling nutritional status, *PreCONUT* preoperative CONUT, *PoCONUT* postoperative CONUT, *HBsAg *hepatitis B viral surface antigen, *AFP* alpha-fetoprotein, *TBIL* total bilirubin, *ALT* alanine aminotransferase, *AST* aspartate minotransferase, *PT* prothrombin time, *CREA* creatinine, *HGB* hemoglobin, *PLT* platelet count, *WBC* white blood cell count, *MVI* microvascular invasion, *HR* hazard ratio, *CI* confidence interval

As for RFS, ten potential covariates with p values less than 0.10 in univariate analysis were further included in Cox regression analysis.Poor differentiation (P = 0.014, HR = 1.588, 95 %CI 1.098–2.298), tumor number (P = 0.049, HR = 1.611, 95 %CI 1.003–2.589), MVI (P = 0.001, HR = 1.963, 95 %CI 1.314 –2.933) and PoCONUT score (P = 0.008, HR = 1.639, 95 %CI 1.139–2.358) were prognostic factors for reduced RFS in multivariate analysis (Table 5).

**Table 5:** Univariate and multivariate analyses of prognostic factors for recurrence-free survival in a propensity score matched cohort

Factors	Univariate analysis		Multivariate analysis	
	HR (95 %CI)	P	HR (95 %CI)	P
Age	0.996 (0.977–1.014)	0.638		
Gender (male)	1.449 (0.855–2.455)	0.168		
HBsAg (+)	1.439 (0.633–3.272)	0.385		
AFP (> 400ng/mL)	1.310 (0.896–1.915)	0.163		
TBIL	0.998 (0.962–1.036)	0.919		
ALT	1.008 (1.001–1.016)	0.030		
AST	1.013 (1.002–1.024)	0.025		
PT	1.093 (0.933–1.280)	0.271		
CREA	1.017 (1.001–1.034)	0.039		
HGB	1.002 (0.988–1.016)	0.832		
PLT	0.997 (0.991–1.003)	0.387		
WBC	0.951 (0.821–1.102)	0.951		
PreCONUT (≥ 3)	0.911 (0.631–1.317)	0.622		
Tumor size (3–5 cm)	0.939 (0.656–1.344)	0.731		
Tumor number (2–3)	1.684 (1.050–2.701)	0.030	1.611 (1.003–2.589)	0.049
Blood loss	1.001 (0.998–1.002)	0.834		
Transfusion (+)	1.167 (0.898–3.110)	0.105		
Cirrhosis (+)	1.352 (0.707–2.585)	0.363		
Operation duration	1.072 (0.864–1.582)	0.461		
Operation type (major)	1.212 (0.889–1.634)	0.573		
MVI (+)	2.169 (1.461–3.221)	< 0.001	1.963 (1.314–2.933)	0.001
Poor differentiation (+)	1.579 (1.095–2.277)	0.014	1.588 (1.098–2.298)	0.014
Hospital stay	1.004 (0.984–1.021)	0.641		
PoCONUT (≥ 3)	1.561 (1.088–2.239)	0.015	1.639 (1.139–2.358)	0.008

*CONUT* controlling nutritional status, *PreCONUT* preoperative CONUT, *PoCONUT* postoperative CONUT, *HBsAg* hepatitis B viral surface antigen, *AFP* alpha-fetoprotein, *TBIL* total bilirubin, *ALT* alanine aminotransferase, *AST* aspartate aminotransferase, *PT* prothrombin time, *CREA* creatinine, *HGB* hemoglobin, *PLT* platelet count, *WBC* white blood cell count; *MVI* microvascular invasion, *HR* hazard ratio, *CI* confidence interval

## Discussion

In the present study, multivariate analysis found that the high PoCONUT score (≥ 3) was an independent prognostic factor for both OS and RFS in patients with small HCC who underwent liver resection. Researchers have established the prognostic value of the PreCONUT score in multiple malignant tumors [12, 20, 21]. To our knowledge, this is the first study to investigate the relationship between the PoCONUT score and the prognosis of patients with small HCC who underwent liver resection using a PSM analysis.

CONUT score was originally used in early detection and continuous control for hospital undernutrition [11]. It is derived from three readily available parameters, serum albumin concentration, total lymphocyte count and total cholesterol concentration. Among them, serum albumin is not only a major indicator of nutritional status but also affected by chronic inflammation, liver function reserve and body fluid change [22, 23]. Hypoalbuminemia was reported to be correlated with immunosuppression and poor prognosis of malignancies [24]. Total cholesterol concentration is another indicator of nutritional status. A decreased concentration of cholesterol indicates not only a calorie deficiency but also that the cell is being deprived of an essential nutrient required to maintain metabolic equilibrium and membrane integrity [25]. And total cholesterol was further reported to be an independent risk factor of poor OS and RFS for patients with HCC [26]. Lymphocyte, another component of the CONUT score, plays a significant role in anti-tumor immunity [27]. Besides, lymphocyte and lymphocyte-related indexes were found to be related to the survival of patients with HCC [28, 29]. Therefore, CONUT score theoretically reflects the immunological and nutritional status of patients with HCC.

Takagi K et al. reported that a PreCONUT score ≥ 3 was a reliable and independent predictor of poor survival after liver resection for patients with HCC in 2017 [16]. Similarly, some other literature found a high PreCONUT score could help to predict the survival of patients with HCC [15, 30]. In the present study, the PreCONUT score had a trend to be related with the survival of HCC patients, but the difference was not statistically significant. We believe this might be caused by the bias of patient selection. In the previous three studies, they enrolled 43 (20.6 %) patients of BCLC tumor stage C, 133 (45.1 %) patients of TNM stage III/IV and 112 (31.4 %) patients of TNM stage III/IV respectively [15, 16, 30]. However, all the patients enrolled in the present study were classified in BCLC tumor stage 0/A. We hypothesized that patients of early tumor stage represented a better immunological and nutritional status when compared to those in mediate/advanced stage. The PreCONUT score may not able to serve as a stable predictor for the survival of patients with small HCC meeting Milan criteria.

In the present study, for the first time, we investigated the clinical value of PoCONUT score, which could reflect the actual immunological and nutritional status after surgical removal of the tumor, and we found PoCONUT score was a stable predictor for both OS and RFS in patients with small HCC after liver resection. In Chinese tradition, a patient would have a very good rest and nutritional support immediately after any kinds of surgery. If a high PoCONUT score emerges with such adequate support, it indicates that the patient’s poor ability and potential to recover from surgery which promotes tumoral recurrence. In the present study, we used a PSM analysis to balance the baseline covariates between high and low PoCONUT groups. It is used in many articles to balance treated groups across all risk factors [12, 19] Interestingly, before PSM, a high PoCONUT score was significantly associated with several host-related factors including higher total bilirubin and PreCONUT score, longer prothrombin time, lower hemoglobin, platelet, and white blood cell counts and higher incidence of liver cirrhosis. These results validated our hypothesis that patients with a high PoCONUT score had a worse potential and ability to recover from surgery.

There is no universally accepted cut-off value of CONUT score for now. High CONUT score was defined as CONUT score ≥ 3 in the present study which was consistent with some previous reports [15, 16]. However, Harimoto N et al. defined the high CONUT score as CONUT score ≥ 4 using a receiver operating characteristic (ROC) analysis [12]. We noticed that the mean age of the study population was lower (49.8 VS 68.6) in the present study. And age is positively related to CONUT score [31]. Only 79 (14.4 %) patients had a PoCONUT score ≥ 4 in the present study. Besides, we tried to identify more patients who were at risk of immunosuppression and undernutrition with a cut-off value of 3. Thirdly, the cut-off value for the PoCONUT score associated with survival using ROC analysis in the present study was also 3 (area under the curve = 0.627, data not shown). Further study is needed to determine the adequate cut-off value of the CONUT score to predict prognosis.

The present study has several limitations. First, this was a single-center, retrospective study, there might be potential selection bias. Second, the predominant etiology in the present study is HBV while most HCC cases are related to hepatitis C Virus and alcohol in western countries and Japan. So, the results of the present study should be further validated by a different etiological population.

## Conclusions

High PoCONUT score helps to predict worse OS and RFS in patients with small HCC who underwent liver resection.

## Data Availability

The datasets generated and analyzed during the current study are not publicly available due to patient privacy but are available from the corresponding author on reasonable request.

## Abbreviations

HCC: Hepatocellular carcinoma
OS: Overall survival
RFS: Recurrence free survival
HR: Hazard ratio
CI: Confidential interval
HBV: Hepatitis B virus
MVI: Microvascular invasion
AFP: Alpha fetal protein
CT: Computed tomography
